# Neonatal sepsis registry: Time to antibiotic dataset

**DOI:** 10.1016/j.dib.2019.104788

**Published:** 2019-11-11

**Authors:** Svetlana Ostapenko, Melissa Schmatz, Lakshmi Srinivasan, Okan U. Elci, Scott L. Weiss, Aaron J. Masino, Marissa Tremoglie, Mary Catherine Harris, Robert W. Grundmeier

**Affiliations:** a Department of Biomedical & Health Informatics, Children's Hospital of Philadelphia, Philadelphia, PA, USA; b Division of Neonatology, Children's Hospital of Philadelphia, Philadelphia, PA, USA; c Department of Pediatrics, Perelman School of Medicine, University of Pennsylvania, Philadelphia, PA, USA; d The Biostatistics and Data Management Core, Children's Hospital of Philadelphia, Philadelphia, PA, USA; e Westat, Rockville, MD, USA; f Department of Anesthesiology and Critical Care, Perelman School of Medicine, University of Pennsylvania, Philadelphia, PA, USA

**Keywords:** Neonatal sepsis, Registries, Anti-bacterial agents, Quality improvement, Infant mortality

## Abstract

This article describes the process of extracting electronic health record (EHR) data into a format that supports analyses related to the timeliness of antibiotic administration. The de-identified data that accompanies this article were collected from a cohort of infants who were evaluated for possible sepsis in the Neonatal Intensive Care Unit (NICU) at the Children's Hospital of Philadelphia (CHOP). The interpretation of findings from these data are reported in a separate manuscript [1]. For purposes of illustration for interested readers, scripts written in the R programming language related to the creation and use of the dataset have also been provided. Interested researchers are encouraged to contact the research team to discuss opportunities for collaboration.

**Table:** Specifications Table

Subject	Critical Care and Intensive Care Medicine
Specific subject area	Sepsis among neonates and infants.
Type of data	Table
How data were acquired	Episodes of possible sepsis among infants in the neonatal intensive care unit (NICU) were identified from electronic health records. Demographic characteristics, co-morbidity status, treatment, and clinical outcome details were extracted electronically. The outcome of the sepsis evaluation (culture positive sepsis, clinical sepsis with negative cultures, non-bacterial infection, or negative for sepsis) was determined by manual chart review.
Data format	Raw
Parameters for data collection	Charts for all infants who had one or more blood cultures collected were reviewed for possible inclusion in the infant sepsis cohort. These charts were reviewed manually to identify and categorize distinct episodes of sepsis evaluation.
Description of data collection	Data were extracted from electronic health records (Epic Systems Inc., Verona, WI) using the structured query language. Candidate episodes of sepsis evaluation were loaded into a REDCap database (Vanderbilt University, Nashville, TN) for subsequent manual review. Demographic, clinical, treatment and outcome data were extracted for sepsis episodes that were marked for inclusion by manual review. Data were re-formatted to the unit of analysis (one row per sepsis evaluation episode) using the R programming language (version 3.5.3).
Data source location	Neonatal Intensive Care Unit, Children's Hospital of Philadelphia, Philadelphia, USA
Data accessibility	Repository name: Neonatal Sepsis Registry: Time to Antibiotic Dataset, Mendeley Data Data identification number: 10.17632/5vdz5cftz7.1 Direct URL to data: https://doi.org/10.17632/5vdz5cftz7.1
Related research article	Melissa Schmatz, M.D., Lakshmi Srinivasan, M.B.B.S., M.T.R., Robert W. Grundmeier, M.D., Okan U. Elci, Ph.D., Scott L. Weiss, M.D., M.S.C.E., Aaron J. Masino, Ph.D., Marissa Tremoglie, B.S., Svetlana Ostapenko, M.S., Mary Catherine Harris, M.D. Surviving Sepsis in the Neonatal Intensive Care Unit: Association between Time to Antibiotic Administration and In-Hospital Outcomes Journal of Pediatrics DOI pending

**Table:** 

**Value of the Data** • These data contain a curated set of information regarding sepsis evaluations among infants in a quaternary neonatal intensive care unit (NICU) and can be used to understand relationships between baseline risk characteristics, timeliness of antibiotic administration, and mortality. • Researchers or quality improvement professionals interested in better understanding the role of timely antibiotic administration and infant mortality may benefit from these data. • The dataset includes information about rates of exposures (e.g. presence of central venous lines), neonatal co-morbidities, and mortality outcomes that may be useful for power or sample size calculations to design future studies related to sepsis among infants. • These data were extracted from electronic health records from a diverse cohort of critically ill infants in an urban quaternary care facility, and were manually reviewed to verify accuracy. The authors welcome opportunities to collaborate and can be contacted to discuss other types of data that may be available for this cohort of infants.

## 1 Data

The dataset linked to this article is a fully de-identified cohort of 1946 sepsis evaluations among 986 infants at Children's Hospital of Philadelphia [2]. Data were collected between September 2014 and February 2018. These data were used to evaluate associations between time to antibiotics, baseline clinical characteristics, and clinical outcomes [1]. The following fields are available in this dataset:

• **episode_id:** Primary key, uniquely identifies each sepsis evaluation episode.
• **unique_patient_id:** Unique pseudo-identifier for each infant. Each infant may have experienced multiple sepsis evaluation episodes
• **sex**: Biological sex, coded as 0 = female and 1 = male
• **race**: Race information provided by the parent at the time of registration, coded as:
  1 = American Indian or Alaska Native
  2 = Asian
  3 = Black or African American
  4 = Native Hawaiian or Other Pacific Islander (not present in dataset)
  5 = White
  6 = Two or more races
  0 = Unknown
• **gestational_age_at_birth_weeks:** Post-menstrual gestation at time of birth, recorded as whole number of weeks
• **birth_weight_kg:** Weight in kilograms as measured at the time of delivery
• **sepsis_group:** Outcome of sepsis evaluation, assigned by manual chart review, coded as:
  1 = positive culture for bacteria from any source, minimum of 5 days (120 hours) of antibiotic treatment
  2 = no positive culture, maximum of 72 hours of antibiotic treatment
  3 = no positive culture, minimum of 5 days (120 hours) of antibiotic treatment (aka “clinical sepsis”)
  4 = positive culture only for viral pathogens (negative for bacterial pathogens)
  5 = positive culture only for fungal pathogens (negative for bacterial or viral pathogens)
  6 = other (e.g. more than 72 hours but less than 120 hours of antibiotic treatment)
• **onset_age_in_days:** Infant age in days of life
• **onset_hour_of_day:** Clock hour of the day (0–23) when sepsis evaluation was initiated
• **blood_culture_positive:** Subset of episodes with culture proven sepsis (sepsis_group = 1) who had culture proven bacteremia (0 = no, 1 = yes)
• **positive_days:** Number of calendar days of positive cultures including the initial day of sepsis evaluation (e.g. a value of “2” indicates that a culture collected on the calendar day after the sepsis evaluation was positive, but any cultures collected thereafter during the episode were negative)
• **cx_site:** Indicates source of positive culture for culture proven sepsis (sepsis_group = 1). Coded as:
  1 = blood
  2 = urine
  3 = pleural or peritoneal fluid
  4 = cerebrospinal fluid
• **time_to_antibiotics:** Number of minutes from initiation of sepsis evaluation to administration of first dose of antimicrobial treatment.
• **stat_abx:** Indicates whether antibiotics were ordered with a priority of “STAT” when the sepsis evaluation was performed (0 = no, 1 = yes)
• **overall_mortality_within_7_days:** Indicates whether the child died within 7 days (168 hours) of sepsis evaluation for any reason (0 = no, 1 = yes)
• **overall_mortality_within_14_days:** Indicates whether the child died within 14 days (336 hours) of sepsis evaluation for any reason (0 = no, 1 = yes)
• **overall_mortality_within_30_days:** Indicates whether the child died within 30 days (720 hours) of sepsis evaluation for any reason (0 = no, 1 = yes)
• **intubated_at_time_of_sepsis_evaluation:** Indicates whether the child was intubated (mechanically ventilated) at the time of sepsis evaluation (0 = no, 1 = yes)
• **intubated_free_days:** Number of days that was not intubated in the 28 days after sepsis evaluation. Intubation status on last day of observation (e.g. for infants who died) was carried forward for the remainder of the 28-day observation period
• **inotrope_at_time_of_sepsis_eval:** Indicates whether the child was receiving inotrope support (pressor medications by continuous infusion) at the time of sepsis evaluation (0 = no, 1 = yes)
• **inotrope_free_days:** Number of days that infant did not receive inotrope support in the 28 days after sepsis evaluation. Inotrope support status on last day of observation (e.g. for infants who died) was carried forward for the remainder of the 28-day observation period.
• **central_venous_line:** Indicates whether a central venous line (e.g. umbilical venous line or peripherally inserted central catheter) was present at the time of sepsis evaluation (0 = no, 1 = yes)
• **umbilical_arterial_line:** Indicates whether an umbilical arterial catheter (UAC) was present at the time of sepsis evaluation (0 = no, 1 = yes)
• **ecmo:** Indicates whether the child was receiving extracorporeal membrane oxygenation (ECMO) treatment at the time of sepsis evaluation (0 = no, 1 = yes)
• **temp_celsius:** Maximum patient temperature in Celsius on calendar day of sepsis evaluation
• **length_of_stay_hours:** Total length of stay from hospital admission to discharge from the NICU, reported in whole number of hours
• **comorbidity_necrotizing_enterocolitis:** Necrotizing enterocolitis at any time before sepsis evaluation (0 = no, 1 = yes)
• **comorbidity_chronic_lung_disease:** Chronic lung disease noted at any time before sepsis evaluation (0 = no, 1 = yes)
• **comorbidity_cardiac:** Complex congenital cardiac disease (0 = no, 1 = yes). Considered to have been present since birth and is either always present or always absent for all sepsis evaluation episodes within a child.
• **comorbidity_surgical:** Complex non-cardiac surgical disease such as congenital diaphragmatic hernia, gastroschisis, spina bifida, encephalocele, etc (0 = no, 1 = yes). Considered to have been present since birth and is either always present or always absent for all sepsis evaluation episodes within a child.
• **comorbidity_ivh_or_shunt:** Presence of intraventricular hemorrhage or ventriculo-peritoneal shunt noted at any time before sepsis evaluation (0 = no, 1 = yes)
• **period:** Year of data collection (1–4)

## 2 Experimental design, materials, and methods

We performed a retrospective analysis of electronic health records for a cohort of infants who were evaluated for sepsis at the Children's Hospital of Philadelphia between September 2014 and February 2018. The electronic health record in use during that time period was the Epic Inpatient product (Epic Systems, Inc., Verona, WI, USA). The following sections describe how data were extracted, cleaned, and formatted to support analyses related to the timeliness of antibiotic administration and mortality among infants with either confirmed or clinical concern for sepsis [1].

### 2.1 Identification of sepsis evaluation episodes

On a daily basis during the data collection period, the EHR vendor's database (Clarity) was queried using structured query language (SQL) to identify blood culture orders for infants admitted to the neonatal intensive care unit (NICU) that were followed within 24 hours by an order for an antibiotic. This list of blood cultures was transferred each day to a REDCap database [3,4]. Research assistants reviewed the list of blood cultures to differentiate actual sepsis evaluations from other artifacts (e.g. erroneous orders, orders that were subsequently cancelled, and cultures that were repeated for an ongoing episode of sepsis). Blood cultures that were “confirmed” as representing a sepsis evaluation were flagged in the REDCap database to trigger assignment of a unique sepsis evaluation identification number and additional data collection. The research assistants also confirmed that the time of sepsis evaluation was correctly attributed to either the blood culture or antibiotic order, whichever occurred first. Finally, they determined the care location (NICU, emergency department, or outside hospital) where the sepsis evaluation was initiated.

### 2.2 Triggering additional data collection

When the research assistants confirmed that a blood culture represented a sepsis evaluation, a REDCap application programming interface (API) sent the sepsis evaluation information (patient identifier, date/time of evaluation, location of sepsis evaluation) back to the EHR vendor's database. Each day a “sepsis data transfer” script transferred a core set of information related to the sepsis evaluation to the REDCap database. This core set of information facilitated quality improvement activities and preliminary analyses for researchers. The core dataset included culture results, selected laboratory values (e.g. complete blood counts), vital signs (e.g. temperature, pulse, blood pressure), type of antibiotics and timing of administration, and child demographic information.

### 2.3 Categorizing sepsis episodes

On a periodic basis after a sufficient follow-up period had elapsed to ensure the outcomes of the sepsis evaluations were known (e.g. culture results and duration of treatment), an analyst ran additional SQL scripts that extracted further information from the EHR database to assign each sepsis evaluation to one of six sepsis groups (see definition of variable “sepsis_group” in prior section). This determination was made based on the final results of cultures that were collected on the day of sepsis evaluation, and the number of days of antibiotic treatment. Infants who died while still receiving antibiotics were categorized as if they had continued antibiotic treatment for at least 120 hours.

### 2.4 Extracting supplemental sepsis information

To support more complex analyses, such as the time to antibiotic administration analyses supported by the dataset described in this article, detailed data from the entire hospitalization for infants who experienced at least one sepsis evaluation were transferred to a PostgreSQL database [5], and transformed into a set of comma separated value (CSV) files in a format based on the Patient-Centered Outcomes Research Network's (PCORnet) common data model [6]. This format was then extended to better accommodate inpatient data of interest to a broader variety of research questions in the manner described by the Pediatric Trials Network (PTN) [7]. This format contains all lab results, vital signs, medication administrations, clinical bedside assessments, diagnoses, and details about the presence of lines, airways, drains and other devices.

### 2.5 Additional program files

The detailed information in the CSV files in the PTN/PCORnet format were then filtered and transformed to create the analytic file for the time to antibiotic (TTA) administration project using scripts in the R programming language [8]. For purposes of illustration of this process, the supporting R program file that transformed data from the PTN/PCORnet format into the key variables that were necessary for the TTA project have been included with this article (nicu-variables.R). Also, the R program to generate the figures for the primary TTA manuscript have been included (tta-sepsis-paper.R) in hopes that it will provide interested readers with additional insight regarding how the TTA dataset can be used.
